# Improved YOLOv8 Model for Lightweight Pigeon Egg Detection

**DOI:** 10.3390/ani14081226

**Published:** 2024-04-19

**Authors:** Tao Jiang, Jie Zhou, Binbin Xie, Longshen Liu, Chengyue Ji, Yao Liu, Binghan Liu, Bo Zhang

**Affiliations:** 1 College of Artificial Intelligence, Nanjing Agricultural University, Nanjing 210031, China; 9213020127@stu.njau.edu.cn (T.J.); 2022819050@stu.njau.edu.cn (J.Z.); 2022819028@stu.njau.edu.cn (B.X.); 9213020527@stu.njau.edu.cn (C.J.); 9213020230@stu.njau.edu.cn (Y.L.); 9213020201@stu.njau.edu.cn (B.L.); 2022819049@stu.njau.edu.cn (B.Z.); 2 Key Laboratory of Breeding Equipment, Ministry of Agriculture and Rural Affairs, Nanjing 210031, China

**Keywords:** pigeon egg detection, YOLOv8, partial convolution, efficient multi-scale attention, exponential moving average

## Abstract

**Simple Summary:**

The utilization of computer vision technology and automation for monitoring and collecting pigeon eggs is of significant importance for improving labor productivity and the breeding of egg-producing pigeons. Currently, research both domestically and internationally has predominantly focused on the detection of eggs from poultry such as chickens, ducks, and geese, leaving pigeon egg recognition largely unexplored. This study proposes an effective and lightweight network model, YOLOv8-PG, based on YOLOv8n, which maintains high detection accuracy while reducing the model’s parameter count and computational load. This approach facilitates cost reduction in deployment and enhances feasibility for implementation on mobile robotic platforms.

**Abstract:**

In response to the high breakage rate of pigeon eggs and the significant labor costs associated with egg-producing pigeon farming, this study proposes an improved YOLOv8-PG (real versus fake pigeon egg detection) model based on YOLOv8n. Specifically, the Bottleneck in the C2f module of the YOLOv8n backbone network and neck network are replaced with Fasternet-EMA Block and Fasternet Block, respectively. The Fasternet Block is designed based on PConv (Partial Convolution) to reduce model parameter count and computational load efficiently. Furthermore, the incorporation of the EMA (Efficient Multi-scale Attention) mechanism helps mitigate interference from complex environments on pigeon-egg feature-extraction capabilities. Additionally, Dysample, an ultra-lightweight and effective upsampler, is introduced into the neck network to further enhance performance with lower computational overhead. Finally, the EXPMA (exponential moving average) concept is employed to optimize the SlideLoss and propose the EMASlideLoss classification loss function, addressing the issue of imbalanced data samples and enhancing the model’s robustness. The experimental results showed that the F1-score, mAP50-95, and mAP75 of YOLOv8-PG increased by 0.76%, 1.56%, and 4.45%, respectively, compared with the baseline YOLOv8n model. Moreover, the model’s parameter count and computational load are reduced by 24.69% and 22.89%, respectively. Compared to detection models such as Faster R-CNN, YOLOv5s, YOLOv7, and YOLOv8s, YOLOv8-PG exhibits superior performance. Additionally, the reduction in parameter count and computational load contributes to lowering the model deployment costs and facilitates its implementation on mobile robotic platforms.

## 1. Introduction

As of today, pigeon farming has rapidly emerged as an industry, with pigeons now recognized as the fourth major poultry species alongside chickens, ducks, and geese [1]. Pigeon eggs are rich in nutrients such as protein, iron, and calcium, and they are easily digestible and absorbable. Consuming pigeon eggs can improve skin quality and blood circulation, and they possess detoxifying properties, making them a high-quality nutritional product [2,3]. However, pigeon eggshells are relatively soft and prone to breakage from being stepped on or pecked by pigeons. In the actual production and breeding process, when the phenomenon of egg laying is detected, pigeon eggs should be immediately placed into electrical incubation equipment and replaced with fake eggs to minimize the impact on the physiological habits of pigeons, ensuring production efficiency and preventing the occurrence of abandoned hatching [4]. Therefore, the rapid and accurate identification of pigeon eggs is particularly important for the management of pigeon eggs.

Currently, both domestically and internationally, there are numerous techniques for detecting various types of poultry eggs using computer vision technology. With the continuous development of technology, certain characteristics and advantages have been formed. In 2008, Pourreza et al. proposed grayscale thresholding of target regions for surface-defect detection on poultry eggs [5]. By comparing the ratio of the projected area after thresholding to the target region with a threshold value, they achieved a detection accuracy of 99%. In 2010, Deng et al. used image-enhancement algorithms to highlight crack features and applied visual detection to egg-crack detection, achieving a high detection accuracy of 98% [6]. In 2012, Lunadei et al. designed a multispectral image-processing algorithm to differentiate stains from normal egg colors, with a recognition speed of 50 ms and a recognition rate of 98% [7]. In 2014, Wang extracted 24 physical parameters for egg-crack detection, achieving a detection accuracy of 96.67% [8]. In 2017, Sunardi et al. [9] applied smartphones, thermal imaging cameras, and MATLAB for poultry egg recognition, achieving a recognition accuracy of 100% [9]. In the same year, Ang et al. proposed a method combining robots to statistically count and collect eggs in free-range chicken farms, with a positioning error within 2 cm [10]. In 2018, Ab Nasir A et al. designed an automatic egg-grading system with positioning and recognition accuracies exceeding 95% [11]. In 2023, Li et al. used an improved YOLOv7 network (MobileOne-YOLO) for detecting fertilized duck eggs, significantly improving FPS performance by 41.6% while maintaining the same accuracy as YOLOv7 [12]. With the development of computer vision and deep learning, the accuracy of many poultry egg-detection models has exceeded 95%, and they perform well in complex background environments. However, most methods only focus on accuracy and do not consider model parameter count and computational load [13].

In 2023, researchers developed machine vision solutions for egg detection on conveyor belts. Huang et al. utilized the CA attention mechanism, BiFPN, and GSConv to enhance YOLOv5 and combined it with byte-tracking algorithms to detect broken eggs [14]. On the other hand, Luo et al. improved YOLOv5 using BiFPN and CBAM to detect leaky eggs [15]. Both Huang and Luo et al. employed different approaches to enhance YOLOv5 for detecting egg defects. It is worth noting that their methods were installed above or at the end of the egg conveyor belt. However, pigeon eggs, with lower shell strength and greater fragility compared to eggs from other poultry such as chickens, ducks, and geese, are not typically transported using conveyor belts. Instead, they are inspected and collected manually, leading to significant manual labor and issues such as egg breakage and embryo death due to prolonged exposure in pigeon coops. To enhance the level of intelligence and automation in pigeon egg breeding, and to increase labor productivity, it is crucial to use pigeon egg-picking robots for detecting egg laying and collecting pigeon eggs. The key to the development of pigeon egg-picking robots lies in the development of precise and efficient pigeon egg-detection algorithms.

Currently, research on egg detection has predominantly focused on poultry such as chickens, ducks, and geese, leaving pigeon egg recognition largely unexplored. Moreover, existing deep learning models based on image and video understanding mostly remain in the research stage. Many existing systems rely on cloud computing models, and few scholars have deployed algorithms on embedded devices to accelerate model deployment. Therefore, this study established a comprehensive database of pigeon eggs from Silver King pigeons, with multiple angles. It distinguished fake pigeon eggs by labeling and specifically adopted C2f-Faster-EMA (CFE) and C2f-Faster (CF) to replace C2f in the backbone and neck networks. Additionally, it introduced the dynamic upsampler Dysample and designed the EMASlideLoss classification loss function to improve the YOLOv8 object-detection algorithm. The proposed YOLOv8-PG algorithm model is efficient and lightweight, making it more suitable for deployment on edge devices and pigeon egg-picking robots, thereby contributing to the scientific nature of egg breeding processes for pigeons and reducing manual costs. Comparison with other common object-detection algorithms in pigeon egg recognition tasks shows that YOLOv8-PG outperforms most models in terms of both accuracy and efficiency.

## 2. Materials and Methods

### 2.1. Data Acquisition and Processing

#### 2.1.1. Data Acquisition

**Figure 1:**
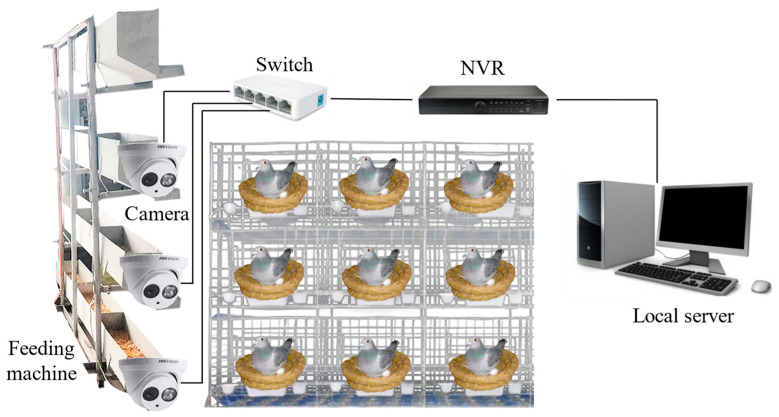
Image data acquisition hardware platform.

From 1 April 2022 to 15 May 2022, video data of pigeon eggs from 150 pairs of breeding pigeons were collected daily. The Silver King pigeon breed was selected as the focus of the study. Custom brackets with Hikvision T12HV3-IA/PoE cameras from Hikvision, Hangzhou, China, were mounted on feeding machines in each row of pigeon coops to capture side-view videos of egg-laying activities. The collected videos were in RGB format. The speed of the cameras was approximately 1 m/min, synchronized with the speed of the feeding machines. Each pigeon coop had a width of 50 cm. Data collection was conducted for 30 min for every batch of 50 pairs of breeding pigeons. A total of 2832 original images were obtained by extracting frames from the collected videos. An illustration of the data collection setup is shown in Figure 1.

#### 2.1.2. Dataset Annotation and Partition

**Figure 2:**
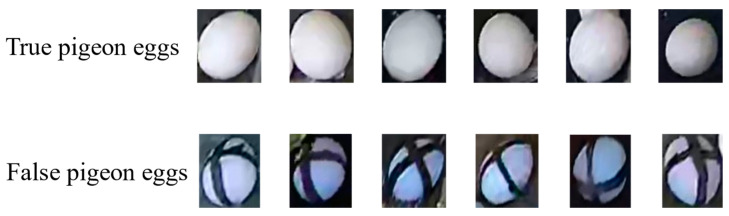
Pigeon egg atlas.

**Figure 3:**
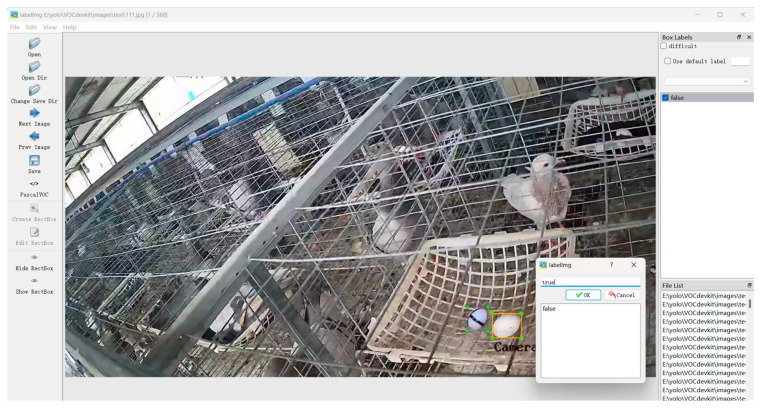
Image annotation interface.

In this study, pigeon eggs were categorized into two categories: real eggs (true) and fake eggs (false). To distinguish fake eggs, they were marked with a black permanent marker to create a cross symbol on their surface. The target extraction of both classes of pigeon eggs was performed on the collected image data, with examples shown in Figure 2. For the detection of real and fake pigeon eggs, the LabelImg image annotation tool was employed to create the COCO dataset, as depicted in Figure 3. A total of 2832 image samples were manually annotated. The training, validation, and testing dataset proportions were set at 8:1:2, resulting in dataset sizes of 1982, 283, and 567, respectively.

Annotation guidelines:

1. All annotated bounding boxes should accurately cover the target, closely fitting the target’s edges in a minimal rectangular box format [16]. They should not exclude any part of the real or fake pigeon eggs, while also avoiding the inclusion of excessive background information.
2. Annotations for real and fake pigeon eggs should maintain consistency and strictly adhere to the requirements of the pigeon egg schema.
3. For partially occluded pigeon egg targets, the annotation should include the occluding object along with the target, ensuring that no clearly identifiable pigeon egg targets are missed.
4. If pigeon eggs are heavily obscured by pigeon cages, feathers, feces, or other breeding pigeons, making them difficult for the human eye to identify, then those targets should not be annotated.

### 2.2. YOLOv8 Network Model

The YOLO (You Only Look Once) series algorithms [17,18,19] are single-stage detection algorithms that balance detection speed with accuracy. YOLOv8, an anchor-free single-stage object-detection algorithm, has become one of the mainstream state-of-the-art (SOTA) models. It supports various computer vision tasks such as object detection, instance segmentation, and object tracking. YOLOv8 offers five scaled versions: YOLOv8n, YOLOv8s, YOLOv8m, YOLOv8l, and YOLOv8x. These versions share the same principles but differ in network depth and width. Considering that the output channel numbers vary across different-scale models, a ratio parameter is used to control the channel count. The overall structure of the network includes the input (receives input images and converts them into a format that the model can process), the backbone network (extracts the target feature from the image), the neck network (realizes image feature fusion), and the detection head network (the output component of the model, responsible for generating inspection results).

**Figure 4:**
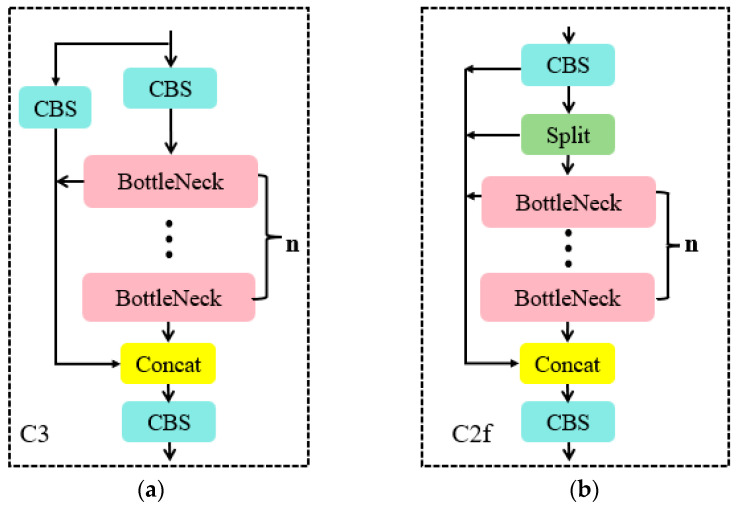
Partial module structure diagram: (**a**) C3, (**b**) C2f.

It is worth noting that, building upon the success of YOLOv5, YOLOv8 introduces new features and improvements to further enhance its performance and flexibility, including [20]:

1. In the backbone network and neck network, YOLOv8 incorporates the design concept of YOLOv7 ELAN [21]. It replaces the C3 structure of YOLOv5 with the C2f structure. The C2f module can combine advanced features with context information to enhance the gradient flow of the model and the feature representation capability of the network by adding additional jump connections, thus improving detection accuracy. The specific module structure is shown in Figure 4.
2. YOLOv8 replaces the detection head with a decoupled-head structure, separating the classification head from the detection head. Additionally, it switches from Anchor-Based to Anchor-Free detection.
3. Regarding the loss function, YOLOv8 separates the regression and classification tasks in object-detection prediction. For the regression task, it employs Distribution Focal Loss (DFL Loss) and Complete Intersection over Union Loss (CIoU Loss). For the classification task, it uses Binary Cross-Entropy Loss (BCE Loss).

### 2.3. YOLOv8-PG Model Improvement Strategy

#### 2.3.1. Fasternet Block

To ensure the network has good detection performance, many improvements have focused on reducing the number of floating-point operations (FLOPs). However, the reduction in FLOPs simultaneously leads to frequent memory access by ordinary convolution operations. In this study, we replaced the Bottleneck in C2f with the Fasternet Block [22] to obtain C2f-Faster, thereby significantly reducing the parameter count and computational load of the network model.

**Figure 5:**
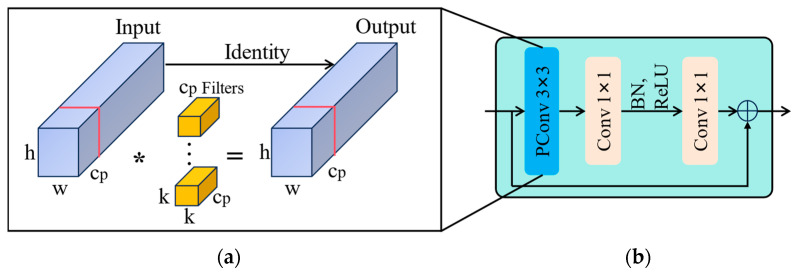
Fasternet Block and its key components: (**a**) Partial convolution, (**b**) Fasternet Block. * stands for convolution operation.

The Fasternet Block utilizes a novel convolutional operation called PConv, which effectively extracts spatial features by reducing redundant computations and memory accesses. PConv applies filters only to a subset of input channels for feature extraction while keeping the remaining channels unchanged. Its structure is illustrated in Figure 5a. Here, h and w represents the height and width of the feature graph, BN represents the batch normalization, and ReLU represents a rectified linear unit.

Assuming the input and output feature maps have an equal number of channels, denoted as c, k represents kernel size and $c_{p}$ represents the number of PConv channels. The FLOPs of PConv are calculated as $h\times w\times k^{2}\times c_{p}^{2}$. When $c_{p}$ is c/4, the FLOPs of PConv become one-sixteenth of those of regular convolution. Additionally, the memory access of PConv is computed as $h\times w\times 2c_{p}+k^{2}\times c_{p}^{2}\approx h\times w\times 2c_{p}$, which is only a quarter of the regular convolution.

Figure 5b illustrates the design of the Fasternet Block module. The Fasternet Block module consists of a PConv and two 1 × 1 Conv layers forming a residual block, with shortcut connections included to reuse important features. Normalization layers and activation layers are only applied after the intermediate 1 × 1 Conv layer, aiming to preserve feature diversity and achieve lower latency.

#### 2.3.2. Efficient Multi-Scale Attention (EMA)

**Figure 6:**
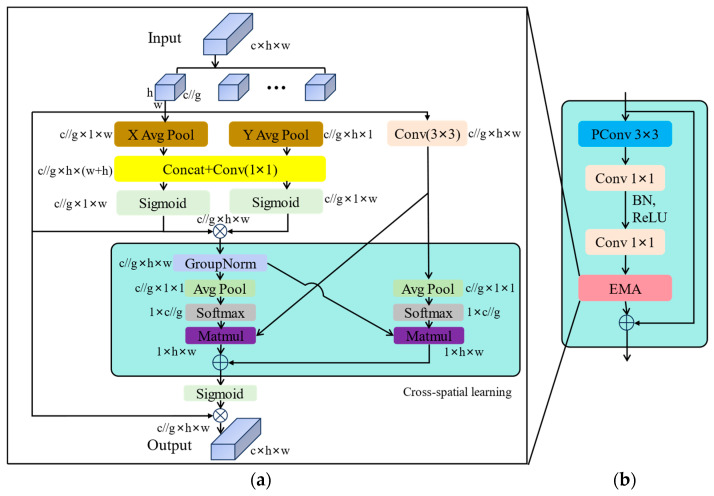
Fasternet-EMA Block and its key components: (**a**) EMA (**b**) Fasternet-EMA Block.

By incorporating attention mechanisms, algorithms can focus more on key areas or features in images and allocate more attention to them, thereby enhancing the performance of the algorithm in object detection. Due to the presence of dust, feces, feathers, and other debris in pigeon coops, there is significant interference with the detection of pigeon eggs, which prevents the original model from fully extracting features. In this study, the EMA attention mechanism [23,24] is introduced into the YOLOv8n network structure to enhance the model’s focus on pigeon egg targets. This enhancement improves the model’s ability to extract spatial features more effectively and reduces interference from the complex environment of pigeon coops. Specifically, by leveraging the flexibility and lightweight nature of EMA, it is incorporated into the Fasternet Block to design the Fasternet-EMA Block, as shown in Figure 6b.

The EMA module does not perform channel downsampling. Instead, it reshapes the channel dimension into a batch dimension using partial channel dimensions, avoiding downsampling through generalized convolution and preventing the loss of feature information. EMA adopts parallel substructures to reduce sequential processing in the network and decrease network depth. The structure of the EMA module is illustrated in Figure 6a. Here, h represents the height of the image, w represents the width of the image, c represents the number of channels in the image, and g represents channel grouping.

Specifically, when presented with specific input feature maps, the EMA attention mechanism initially divides them into G sub-feature maps along the channel dimension to facilitate learning of different semantic features. The EMA module consists of three parallel branches, with two parallel branches located in the 1 × 1 branch and the third branch in the 3 × 3 branch. The 1 × 1 branch utilizes two one-dimensional global average pooling operations to encode channels along two spatial directions, while the 3 × 3 branch stacks single 3 × 3 kernels to capture multi-scale feature representations.

#### 2.3.3. Dysample

In object-detection tasks, upsampling operations are required to adjust the size of input feature maps to match the dimensions of the original image, allowing the model to effectively detect objects of various sizes and distances. Traditional upsampling methods typically rely on bilinear interpolation [25]. These methods have inherent limitations and may result in the loss of crucial image details. Moreover, traditional kernel-based upsampling processes entail a significant amount of computation and parameter overhead, which is not conducive to achieving lightweight network architectures [26]. In real-world pigeon-coop scenarios, pigeon egg images are relatively small, and issues such as pixel distortion may occur, leading to the loss of fine-grained details and difficulty in learning features during recognition tasks. To address this issue, this paper introduces Dysample [27], a highly lightweight and effective dynamic upsampler, aimed at enhancing the detection capabilities for low-resolution images or smaller pigeon egg targets, while reducing instances of false positives and false negatives. Dysample utilizes a point-based sampling method and a perspective of learning sampling for upsampling, completely avoiding time-consuming dynamic convolution operations and additional sub-networks. It requires fewer computational resources and can enhance image resolution without adding extra burden, thus improving model efficiency and performance with minimal computational cost. 

**Figure 7:**
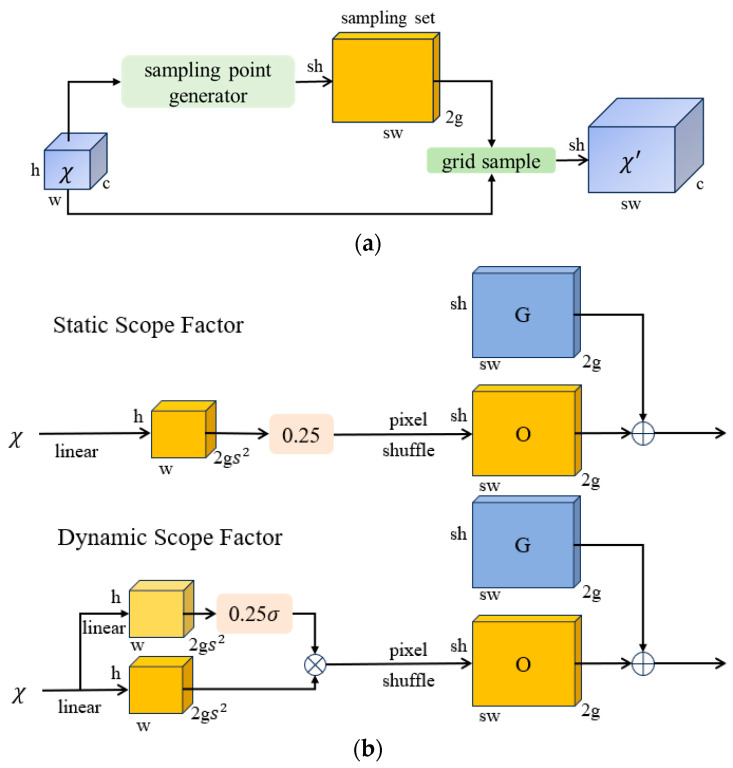
Dysample network structure: (**a**) Sampling based dynamic upsampling; (**b**) Sampling point generator in DySample. The input feature, upsample feature, generated offset, and original grid are denoted by χ, $\chi^{\prime }$, G and O, respectively. σ denotes the sigmoid function. sh represents the sampled height, sw represents the sampled width. $gs^{2}$ represents the number of channels after the feature graph passes through the linear layer.

The network structure of Dysample is illustrated in Figure 7. Its sampling set S consists of the original sampling grid (O) and generated offsets (G). The offsets are generated using a “linear + pixel shuffle” method, where the range of offsets can be determined by static and dynamic factors. Specifically, taking the static factor sampling method as an example, given a feature map of size c×h×w and an upsampling factor s, the feature map first passes through a linear layer with input and output channels of c and ${2s}^{2}$, respectively. Then, it is reshaped using the pixel shuffle method into 2×sh×sw, where 2 represents the x and y coordinates. Finally, the upsampled feature map of size c×sh×sw can be generated.

#### 2.3.4. EMASlideLoss

The problem of sample imbalance, wherein the quantity of easy samples is often significantly larger than that of difficult samples, has garnered widespread attention. In the SlideLoss loss function [28], the Intersection over Union (IoU) value between predicted boxes and ground truth boxes is utilized as an indicator to distinguish between easy and difficult samples. Due to the limited ability to discern difficult samples, the network model cannot effectively utilize the data during training. The function f(x) is employed to assign a higher weight to difficult samples and a lower weight to easy samples, thereby ensuring that the loss function pays more attention to difficult samples. The allocation rule function is as follows:

(1) $$\;f\left(x\right)=\left\{\begin{array}{c} 1\;\;,\;x\leq \mu -0.1\\ e^{1-\mu },\;\mu -0.1<x<\mu \\ e^{1-x}\;\;,\;x\geq \mu \end{array}\right.$$

The function f(x) represents the sliding function operation;x denotes the Intersection over Union (IoU) between predicted boxes and ground truth; and μ represents the weight threshold. 

In specific, the SlideLoss method utilizes the average IoU value of all bounding boxes as the threshold μ, considering values below μ as negative samples and values above μ as positive samples. In this study, we employ the concept of exponential moving average (EXPMA) [29] to optimize the parameter μ within the model. The specific calculation formula is as follows:

(2) $$\mu_{t}=\beta \times \mu_{t-1}+\left(1-\beta \right)\times \theta_{t}$$

where ${\;\theta }_{t}$ represents all parameter weights obtained in the t-th update and $\mu_{t}$ denotes the moving average of all parameters in the t-th update. β denotes the weight parameter.

The sliding average can be regarded as the average value of a variable over a certain period of time. Compared to direct assignment, the value obtained through sliding average is smoother and less jittery in the graph, and it does not fluctuate significantly due to occasional abnormal values. The sliding average can enhance the robustness of the model on test data. Although the dataset used in this study is already quite extensive and rich, there is still a shortage in using these data to train the model due to the relatively small number of difficult samples. Therefore, this study proposes the EMASlideLoss loss function, which optimizes the SlideLoss function using the exponential moving average (EXPMA) approach to address the issue of sample imbalance and enhance the robustness of the model.

### 2.4. YOLOv8-PG Network Model

**Figure 8:**
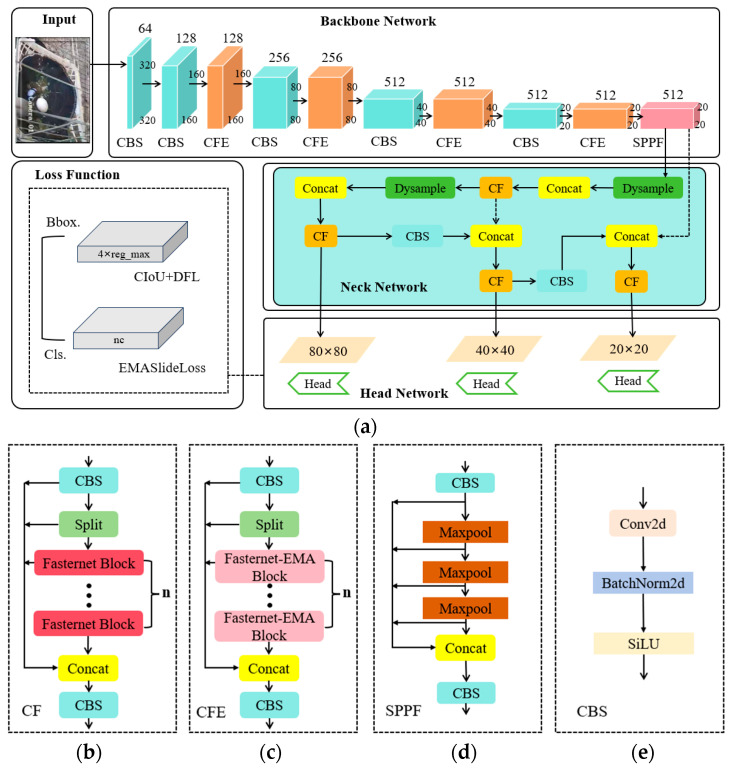
YOLOv8-PG overall framework and its constituent modules: (**a**) YOLOv8-PG, (**b**) C2f-Faster, (**c**) C2f-Faster-EMA, (**d**) Spatial Pyramid Pooling Fusion, (**e**) Conv-BN-SiLU.

In summary, this paper has made improvements to the YOLOv8n model in the four aspects mentioned above. The overall network structure of the improved YOLOv8-PG model is illustrated in Figure 8. Specifically, the C2f-Faster-EMA module was designed for the backbone network, and the C2f-Faster module was used to replace the C2f module in the neck network and introduced the Dysample to the neck network. Regarding the loss function, the EMASlideLoss classification loss function was designed.

## 3. Experiments and Results

### 3.1. Experimental Details

The model for detecting real and fake pigeon eggs was trained based on the PyTorch framework [30]. The training was conducted for 300 epochs with a batch size of 16. The initial learning rate was set to 0.01, and the weight decay coefficient was set to 0.0005. A warm-up training strategy was employed with warm-up epochs set to 3 and warm-up momentum set to 0.8. To reduce memory usage and improve training speed, mixed precision training strategy was adopted. Additionally, mosaic image augmentation was disabled in the last 10 epochs. For detailed experimental environment and hyperparameter settings, please refer to Table 1 and Table 2, respectively.

### 3.2. Model Evaluation Index

To better evaluate the performance of the model, this experiment adopts the following metrics: F1-score (F1), mean average precision (mAP), model parameters (Params), and giga floating-point operations per second (GFLOPs). The specific calculation formulas are as follows:

(3) $$P=\frac{TP}{TP+FP}\times 100\%$$

(4) $$R=\frac{TP}{TP+FN}\times 100\%$$

(5) $$F1=\frac{2\times P\times R}{P+R}\times 100\%$$

(6) $$AP=\int_{0}^{1}P\left(R\right)dR\times 100\%$$

(7) $$mAP=\frac{1}{n}\sum_{k=1}^{k=n}AP_{k}\times 100\%$$

Among them, n index is the number of detection types, and two types of objects are detected in this experiment. TP represents the number of correct detections, and FP indicates the number of errors detected. FN indicates the number of missed tests, and F1 represents the harmonic mean of precision and recall with the confidence threshold is 0.5. AP represents the accuracy of a certain category, and mAP represents the average accuracy for all categories.

The metrics mAP50 and mAP75 correspond to different thresholds of Intersection over Union (IoU). Specifically, mAP50 is calculated using an IoU threshold of 0.50, while mAP75 uses a threshold of 0.75. On the other hand, mAP50-95 refers to the mean average precision calculated over a range of IoU thresholds from 0.50 to 0.95, with increments of 0.05. Generally, the mAP50-95 index is the most stringent as it considers a wider range of IoU thresholds, followed by mAP75, while mAP50 has the lowest threshold and less-strict requirements.

### 3.3. Ablation Experiment Results

To verify the effectiveness of each improvement on the YOLOv8n algorithm, we conducted ablation experiments on the test set of the same pigeon egg dataset, based on the original YOLOv8n model. The ablation experiments are as follows: A: Replaced C2f with C2f-Faster-EMA in the backbone network to reduce the interference of complex environments on pigeon egg detection. B: Replaced C2f in the neck network with C2f-Faster for lightweight processing. C: Improved the upsampling in the neck network using Dysample to enhance the detection ability of low-resolution and small target pigeon eggs. D: Replaced the loss function with EMASlideLoss to enhance the robustness of the model. The experimental results are shown in Table 3.

Table 3 shows:

(1) The first group presents the experimental results of the baseline YOLOv8n, serving as the benchmark for the following four groups of experiments. Its F1 is 97.54%, and mAP50, mAP75, and mAP50-95 are 99.19%, 85.01%, and 68.86%, respectively. The parameter count is 3.006 M, and the GFLOPs value is 8.1 G.
(2) The experiments from the second group to the fifth group progressively incorporate one improvement point at a time. After replacing the Bottleneck of C2f with the Fasternet-EMA Block module in the backbone network of YOLOv8n, the model’s accuracy in various aspects improved, and both the computational complexity and parameter count decreased. Further introducing Fasternet Block into the neck network C2f results in a slight decrease in accuracy, but the model becomes more lightweight, with a reduction of 0.7 G in parameters and 0.344 M in computations. After introducing the ultra-lightweight and effective dynamic upsampler Dysample, F1, mAP50-95, and mAP75 all improved, with negligible impact on parameters and computations. Finally, replacing the EMASlideLoss classification loss function does not increase the model’s parameter count or computational complexity but improves the imbalance of samples, leading to a further improvement in model accuracy.
(3) The fifth group shows the results of adding all improvement points. Compared to the baseline model, the YOLOv8-PG model increased F1 by 0.76%, and the mAP50, mAP75, and mAP50-95 metrics improved by 0.14%, 4.45%, and 1.56%, respectively. Additionally, the computational complexity significantly decreased, with GFLOPs reduced from 8.1 G to 6.1 G and parameters reduced from 3.006 M to 2.318 M, representing reductions of 24.69% and 22.89%, respectively.

### 3.4. Experimental Comparison with Other Models

To validate the superiority of the proposed algorithm, this study conducted comparative experiments with a series of object-detection algorithms, including Faster R-CNN, YOLOv5s, YOLOv7, YOLOv8n, and YOLOv8s. The experimental results demonstrate that the proposed algorithm achieves an mAP50 of 99.33%, surpassing Faster R-CNN by 9.86%. For the more stringent mAP75 metric, the proposed algorithm outperforms YOLOv5s, YOLOv8n, and YOLOv8s by 1.22%, 4.45%, and 2.06%, respectively. Compared to the YOLOv7 model, the proposed algorithm’s mAP50-95 shows a slight decrease of 0.26%, but its parameter count and computational load are only 6.353% and 6.201% of the YOLOv7 model, respectively. The comparative experimental results of the algorithms are presented in Table 4.

**Figure 9:**
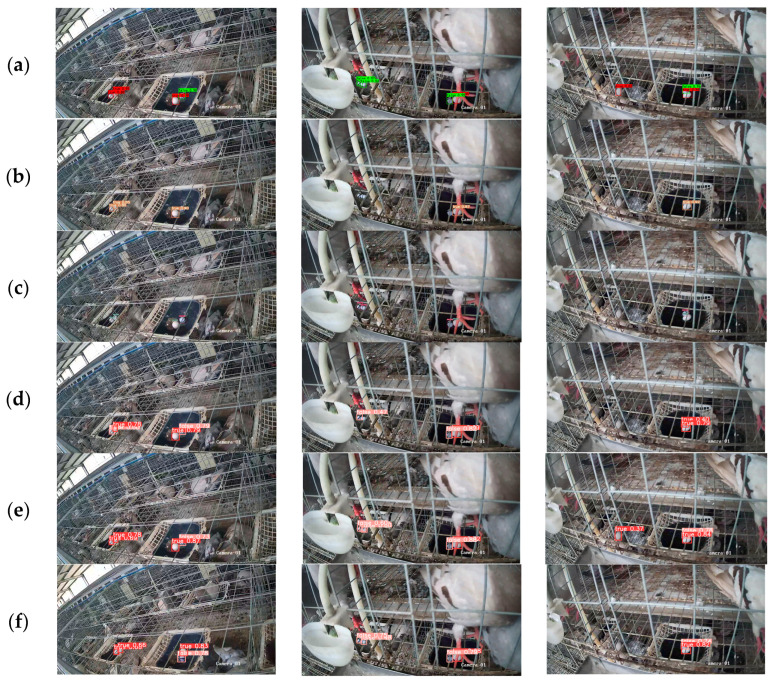
Real and fake pigeon egg-detection results of different models: (**a**) Faster R-CNN (**b**) YOLOv5s (**c**) YOLOv7 (**d**) YOLOv8n (**e**) YOLOv8s (**f**) YOLOv8n-PG.

The experimental results demonstrate that the optimized YOLOv8n model proposed in this study maintains high detection rates and accuracy while reducing memory overhead and detection time. It consumes fewer memory resources compared to the original YOLOv8 model and mainstream object-detection algorithms, making it suitable for deployment on mobile terminals or mobile robotic platforms. Figure 9 visualizes the detection results of real and fake pigeon eggs for different models.

### 3.5. Model Improvement Visualization

**Figure 10:**
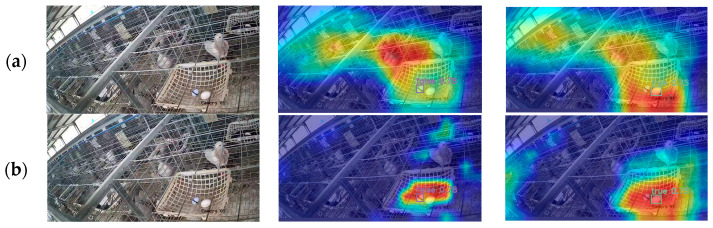
Comparison of thermal map visualization results: (**a**) YOLOv8n (**b**) YOLOv8-PG. Colors represent scalars of one order of magnitude, with hot tones (such as red or yellow) representing areas of high activity or importance, and cool tones (such as blue or green) representing areas of low activity or importance.

The heatmap is a visualization technique used in object detection, which can display the distribution of intensity of the objects detected by the model in the input image. Brighter areas indicate higher attention from the model. To visually demonstrate the optimization effect of the proposed YOLOv8-PG model on the real and fake pigeon egg dataset, this study employs the Grad-CAM [31] (Gradient-weighted Class Activation Mapping) algorithm for visual analysis. Partial detection results before and after algorithm improvement are shown in Figure 10.

From Figure 10, it can be observed that the pigeon farm environment is complex. When YOLOv8n is used for the detection of real and fake pigeon eggs, the background area introduces noise to the model, causing it to focus on some background areas with weaker focus. However, after adding the attention module to improve the model, the noise from the background area is significantly reduced. The model can accurately focus on the pigeon egg area, effectively enhancing the model’s ability to extract features of pigeon egg targets in complex environments.

**Figure 11:**
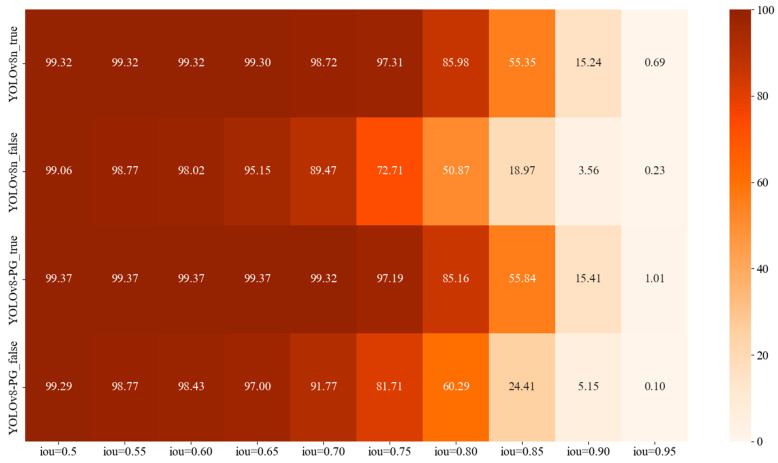
AP value heat map of each detection target.

Figure 11 illustrates the detection effects of YOLOv8n and YOLOv8-PG models on real and fake pigeon eggs under different IoU thresholds—that is, AP values of each category, presented in the form of heat maps. With the increase in IoU, the AP value of each category decreased gradually. When the IoU threshold is lower than 0.8, the AP value of both models is higher than 85% for the real pigeon egg target. The results show that the two models can detect the real pigeon eggs well. For fake pigeon eggs, when the IoU is 0.7 to 0.8, the pigeon eggs are sticky and occluding seriously, and the YOLOv8n model cannot be effectively detected, while the YOLOv8-PG model can reduce the interference of the complex environment and the detection accuracy is increased by 2.3%, 9%, and 9.42%, respectively.

## 4. Discussion

Since the proposal of detection models based on deep learning, they have been widely applied in various industries, with researchers making great efforts to design suitable models and continuously optimize them. Commonly used object-detection methods can be roughly divided into two categories: single-stage detectors and two-stage detectors. Xu et al. [32] deployed an improved Mask R-CNN, a two-stage detector, on mobile robots for egg detection, achieving a high accuracy of 94.18%, but it had a slow detection speed of only 0.76 FPS. Due to not requiring a proposal generation stage, single-stage detectors can obtain detection results in a single pass, often resulting in higher processing speeds compared to two-stage detectors. With the iterations of the YOLO series of single-stage detectors, YOLOv8 utilizes anchor-free and decoupled heads to independently process objects, allowing each branch to focus on its own task. YOLOv8 prioritizes the balance between speed and accuracy—a crucial consideration for integrated system applications. Furthermore, YOLOv8 is more friendly towards detecting small objects, making it the chosen baseline model for our research.

Research has shown that incorporating attention mechanisms and introducing upsamplers [33,34,35,36] have been proven to effectively enhance model detection accuracy. Zeng et al. [37] proposed a YOLOv8 model based on the CBAM mechanism, which can effectively select key features of targets, achieving high-precision recognition of coal and gangue. Li et al. [38] proposed an algorithm based on an improved YOLOv5s to achieve target detection and localization in tomato picking. This algorithm replaces upsampling with the CARAFE structure, which improves network sensitivity and accuracy while maintaining lightweightness. It is worth mentioning that different application environments and datasets require the customization, modification, and development of models based on single-stage detectors (such as YOLO). Even if the working principle of the model remains unchanged, meaningful architectural modifications must be proposed to perform sufficient customization of deep learning models. Considering the complex environment of pigeon coops and the deployment requirements of models, we improved YOLOv8 using C2f-Faster-EMA, C2f-Faster, and Dysample to reduce the interference of complex environments on the model and enhance the model’s ability to detect low-resolution and small targets. Wang et al. [39] addressed the problem of imbalanced difficulty samples by introducing SlideLoss, but they used a fixed value as the threshold for discriminating difficult samples, which cannot improve the model’s generalization ability. We optimized the threshold using exponential moving average (EXPMA) and proposed the EMASlideLoss loss function, effectively improving model performance and enhancing model robustness. Combining the above four improvements, this study proposes the YOLOv8-PG model, with F1, mAP50-95, and mAP75 values of 98.3%, 70.42%, and 89.46%, respectively, indicating that the model effectively recognizes real and fake pigeon eggs. The parameters and computation amount of the model are 2.318 M and 6.4 G, respectively, indicating that the model architecture is lightweight and has the potential to be deployed on embedded devices or mobile platforms.

In recent years, scholars have conducted research on model deployment. Ju et al. [40] proposed the MW-YOLOv5s rice-recognition model and successfully deployed it on a weeding robot, meeting the practical requirements for both detection accuracy and speed. Yu et al. [41] utilized strategies such as SPD-Conv, CARAFE, and GSConv to propose the lightweight model SOD-YOLOv5n, with a model size of only 3.64 M. They successfully deployed the model on Android devices for the real-time detection and counting of winter jujubes. In the future, we plan to deploy the YOLOv8-PG model on robots for automated egg detection. However, the model still has some limitations. Firstly, the data collection method in this study involved installing cameras on feeders, resulting in a high installation height and wide field of view. However, the actual perspective of future intelligent egg-picking robots may be limited and more prone to occlusion issues. Secondly, the real and fake pigeon egg dataset in this study only collected images of pigeon eggs from the Silver King breed, which may introduce biases compared to eggs from other breeds. Therefore, in future research, we will further expand the dataset by adding images of pigeon eggs from different angles and different breeds to enhance the model’s robustness. Additionally, to deploy the trained model on actual robots, this study will consider converting the trained PyTorch format weights to formats such as ONNX and TensorRT. Furthermore, we will utilize TensorRT for acceleration and test the model’s performance on edge devices such as Jetson and Raspberry Pi using methods such as FP16 and INT8 quantization.

## 5. Conclusions

This paper addresses the high rate of pigeon egg breakage and the high cost of manual labor in pigeon egg breeding by proposing a pigeon egg-detection algorithm called YOLOv8-PG, based on a YOLOv8 design. This algorithm is capable of detection in complex environments with limited computational resources. Firstly, by combining the Fasternet Block with the EMA attention mechanism and introducing them into the backbone network of the algorithm, the model’s feature-extraction capability for pigeon egg targets is enhanced. Secondly, introducing C2f-Faster into the neck network of the algorithm further reduces the weight of the model, reducing the model’s parameter and computational complexity. Additionally, the dynamic upsampler Dysample, based on point sampling, is introduced into the model to enhance its detection capability for low-resolution and small-target objects with minimal computational overhead. Finally, the SlideLoss loss function is optimized using the EXPMA concept, and EMASlideLoss is proposed to address the problem of sample imbalance, enhance model robustness, and improve algorithm performance.

The experimental results demonstrate that compared to the two-stage algorithm Faster R-CNN, the model designed in this study shows significant competitiveness in terms of detection accuracy. Compared to other YOLO algorithms in the same series, YOLOv8-PG reduces memory overhead and detection time while maintaining a high detection rate and accuracy. Relative to the baseline YOLOv8n, the YOLOv8-PG model shows improvements in the F1 score by 0.76% and the mAP50, mAP75, and mAP50-95 metrics by 0.14%, 4.45%, and 1.56%, respectively. Additionally, there is a significant reduction in computational complexity, with GFLOPs decreasing from 8.1 G to 6.1 G and parameters decreasing from 3.006 M to 2.318 M, representing reductions of 24.69% and 22.89%, respectively. The improved YOLOv8-PG model, incorporating various methods, demonstrates the potential for integration into pigeon egg-picking robots, with satisfactory robustness and applicability in terms of detection accuracy and model deployment feasibility.

## Figures and Tables

**Table 1:** Experimental environment.

Software Environment	Hardware Environment
Operating system: Ubuntu 20.04	CPU: 15 vCPU AMD EPYC 7742 64-Core
Programming language: Python 3.8	GPU: RTX 3090 (24 GB) × 1
Deep learning framework: PyTorch 2.0.0	
Accelerated environment: CUDA 11.8	

**Table 2:** Hyperparameter Settings.

Parameters	Values
lr0	0.01
lrf	0.01
Momentum	0.937
Weight decay	0.0005
Warmup epochs	3
Warmup momentum	0.8
Batch size	16
Epochs	300
Workers	8

**Table 3:** Comparison results of ablation experiments of different models.

	A	B	C	D	F1	mAP50-95	mAP75	mAP50	Params	GFLOPs
1	×	×	×	×	97.54	68.86	85.01	99.19	3.006 M	8.1 G
2	√	×	×	×	97.81	69.45	86.67	99.01	2.649 M	7.1 G
3	√	√	×	×	98.07	69.10	85.97	99.23	2.305 M	6.4 G
4	√	√	√	×	98.17	69.94	88.57	99.27	2.318 M	6.4 G
5	√	√	√	√	98.30	70.42	89.46	99.33	2.318 M	6.4 G

Note: “√” represents the model’s usage of a particular module, while “×” indicates that the model does not utilize that module. A: Replaced C2f with C2f-Faster-EMA in the backbone network; B: Replaced C2f with C2f-Faster in the backbone network; C: Introduce Dysample in the neck network; D: Replaced the loss function with EMASlideLoss.

**Table 4:** Performance comparison between various network models.

Model	F1	mAP50-95	mAP75	mAP50	Params	GFLOPs
Faster R-CNN	68.98	42.70	32.35	89.47	136.710 M	369.7 G
YOLOv5s	98.80	69.15	88.24	99.47	7.057 M	16.3 G
YOLOv7	98.83	70.68	90.07	99.63	36.487 M	103.2 G
YOLOv8n	97.54	68.86	85.01	99.19	3.006 M	8.1 G
YOLOv8s	97.63	69.2	87.41	99.25	11.136 M	28.4 G
YOLOv8-PG	98.3	70.42	89.46	99.33	2.318 M	6.4 G

## Data Availability

The data presented in this study are available on request from the corresponding author.

## Abbreviations

The following abbreviations are used in this manuscript:

EMA	Efficient Multi-scale Attention
PConv	Partial Convolution
EXPMA	Exponential Moving Average
EMASlideLoss	Exponential Moving Average Slide Loss
Faster R-CNN	Faster Region-based Convolutional Neural Networks
Mask R-CNN	Mask Region-based Convolutional Neural Networks
YOLO	You Only Look Once
MATLAB	Matrix Laboratory
CA	Coordinate Attention
CBAM	Convolutional Block Attention Module
BiFPN	Bidirectional Feature Pyramid Network
SOTA	state-of-the-art
ELAN	Efficient Layer Attention Network
CIoU Loss	Complete Intersection over Union Loss
DFL Loss	Distribution Focal Loss
BCE Loss	Binary Cross-Entropy Loss
IoU	Intersection over Union
Conv	Convolution
SiLU	Sigmoid Linear Unit
BN	Batch Normalization
SPPF	Spatial Pyramid Pooling-Fast
FLOPs	floating point operations
GFLOPs	Giga Floating-point Operations Per Second
P	Precision
R	Recall
F1	F1-score
AP	Average Precision
mAP50	mean Average Precision at IoU threshold of 0.5
mAP75	mean Average Precision at IoU threshold of 0.75
mAP50-95	mean Average Precision at a range of IoU thresholds from 0.50 to 0.95
FPS	Frame Per Second
Grad-CAM	Gradient-weighted Class Activation Mapping
